# Dietary and Sentinel Factors Leading to Hemochromatosis

**DOI:** 10.3390/nu11051047

**Published:** 2019-05-10

**Authors:** Chang-Kyu Oh, Yuseok Moon

**Affiliations:** 1 Laboratory of Mucosal Exposome and Biomodulation, Department of Biomedical Sciences, Pusan National University, Yangsan 50612, Korea; a_x_is@hanmail.net; 2 BioMedical Research Institute, Pusan National University, Yangsan 50612, Korea; 3 Program of Food Health Sciences, Busan 46241, Korea

**Keywords:** hemochromatosis, iron transport and metabolism, stress sentinel

## Abstract

Although hereditary hemochromatosis is associated with the mutation of genes involved in iron transport and metabolism, secondary hemochromatosis is due to external factors, such as intended or unintended iron overload, hemolysis-linked iron exposure or other stress-impaired iron metabolism. The present review addresses diet-linked etiologies of hemochromatosis and their pathogenesis in the network of genes and nutrients. Although the mechanistic association to diet-linked etiologies can be complicated, the stress sentinels are pivotally involved in the pathological processes of secondary hemochromatosis in response to iron excess and other external stresses. Moreover, the mutations in these sentineling pathway-linked genes increase susceptibility to secondary hemochromatosis. Thus, the crosstalk between nutrients and genes would verify the complex procedures in the clinical outcomes of secondary hemochromatosis and chronic complications, such as malignancy. All of this evidence provides crucial insights into comprehensive clinical or nutritional interventions for hemochromatosis.

## 1. Introduction: Regulation of Dietary Iron Metabolism

**Figure 1:**
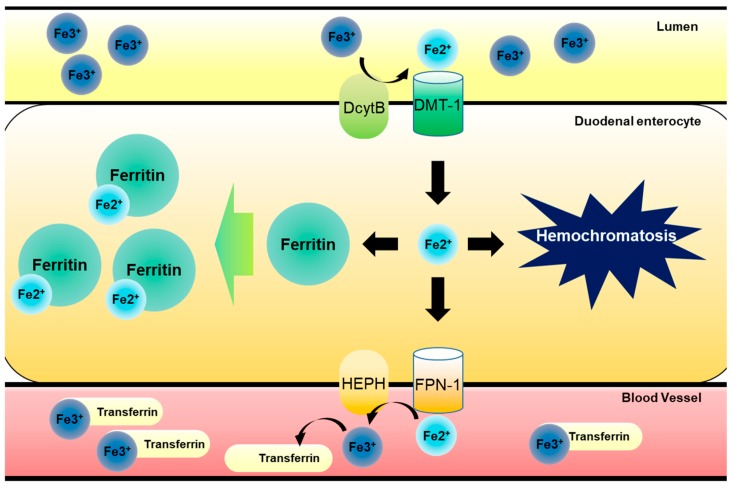
Dietary iron metabolism in gut. Luminal ferric Fe (III) ion from food intake is reduced to ferrous Fe (II) ion by DcytB in apical site of duodenal epithelia. After this, reduced ferrous Fe (II) ion is transported into gastrointestinal enterocytes by DMT-1. Imported ferrous Fe (II) ion have two pathways of modulation. One involves its binding to ferritin, which can store iron ions safely, preventing radial iron ion production, and another pathway involves FPN-1-mediated transport to bloodstream. Exported ferrous Fe (II) ion is then oxidized to ferric Fe (III) ion by HEPH in basolateral site of duodenal epithelial cells. Oxidized ferric Fe (III) ion binds to transferrin circulating bloodstream and is transported to bone marrow for erythrocytosis or liver for storage. However, free ferrous Fe (II) ions can accumulate in cells due to inhibition of the export to bloodstream or increase in import, leading to hemochromatosis.

Iron is an essential metal nutrient required for all living organisms [1,2]. In response to various cues, including iron deficiency in the body, dietary iron ions can be absorbed in the apical site of duodenal enterocyte by two types of membrane proteins. One is duodenal cytochrome B (DcytB), which can reduce ferric Fe (III) ion to ferrous Fe (II) ion for transporting into the enterocytes. Another is divalent metal transporter-1 (DMT-1), which transports divalent metal ions into cells as the name implicates. Imported ferrous Fe (II) ions need to be intracellularly stored by binding to ferritin, due to the toxicity of the free form [3]. However, the stored irons can be released from enterocytes, which is facilitated by ferroportin (FPN, an iron transporter) and a ferroxidase such as hephaestin (HEPH) or ceruloplasmin on the basolateral membrane of duodenal enterocyte in response to signals from the deficient organs, including bone marrow. Exported ferrous Fe (II) ions are oxidized to ferric Fe (III) ion by HEPH and the oxidized ferric Fe (III) ion in a complex with transferrin is delivered via blood circulation to the target organs, such as the bone marrow for erythrocytosis, or liver for storage [4]. However, when stored, ferrous Fe (II) iron cannot be exported to blood vessels for various reasons and it accumulates in cells and organs, leading to iron overload (also known as hemochromatosis) in susceptible tissues, including the gut and liver (Figure 1). 

**Figure 2:**
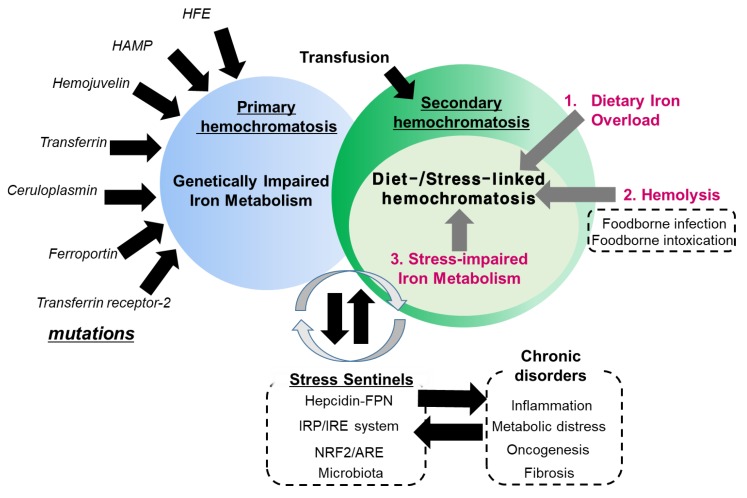
Etiological network in secondary hemochromatosis. Primary hemochromatosis is associated with mutation in genes involved in iron transport and metabolism, including HFE, hepcidin antimicrobial peptide (HAMP), hemojuvelin, transferrin, ceruloplasmin, ferroportin and transferrin receptor-2. Conversely, secondary hemochromatosis is linked to exposure to excess amounts of iron by transfusion or diet-associated etiologies including dietary iron overload via consumption of high iron-containing food, hemolysis-linked iron overload via foodborne factors (infection and intoxication), and stress-impaired iron metabolism. In particular, stress-impaired iron metabolism is closely associated with the stress responsive sentinels which are involved in the susceptibility to the hemochromatosis and other chronic distress. Some mutations in the sentinel-linked genes contribute to primary hemochromatosis.

Hemochromatosis in the liver, heart and endocrine glands has been associated with the triggering of liver cirrhosis, diabetes, cardiomyopathy or testicular failure [5,6,7,8]. In particular, genetic abnormalities in the iron transporting- or storage-linked genes may cause severe disorders of iron homeostasis and subsequent iron overload in most patients with primary hemochromatosis (also known as hereditary hemochromatosis) [5,6,7,8]. Most hereditary hemochromatosis is due to a mutation of the *HFE* gene, which can regulate iron uptake by interfering with the interactions between transferrin and transferrin receptor [9]. However, the primary action of HFE protein is the regulation of the iron storage hormone hepcidin [10], suggesting that it is involved in regulating systemic iron absorption rather than local iron uptake mediated by transferrin receptor. Moreover, hereditary hemochromatosis can be produced by other mutations in iron-modulating genes, such as hemojuvelin, hepcidin antimicrobial peptide (HAMP), transferrin receptor-2, ferroportin, ceruloplasmin and transferrin [11,12,13]. All of the genetic evidences in hereditary hemochromatosis provide insights into functions of iron metabolism-involved components in hemochromatosis. Conversely, there has been considerable clinical debate about whether hemochromatosis should be defined by genotype or presence of symptomatic iron excess independent of genotype [14,15]. There is non-mutagenic hemochromatosis, which is also called secondary hemochromatosis. Secondary hemochromatosis is mostly due to intended or unintended iron exposure to the body or the iron overload due to stress-impaired iron metabolism, which has not been well-addressed [16,17]. The potential causes of the systemic iron overload are transfusion, dietary iron excess, iron poisoning, massive hemolysis, ineffective erythropoiesis and underlying diseases, such as liver cirrhosis, steatohepatitis and porphyria cutanea tarda [17,18,19,20]. Transfusion has been well-addressed as a main cause of systemic iron overload. Repetitive transfusions within a short period of time lead to an accumulation of red blood cells (RBC), subsequent extraordinary burden of disrupted RBCs and subsequent release of heme with ferrous Fe (II). This acute overload from heme-bound iron can predispose a person to hemochromatosis and subsequent iron poisoning in severe cases [21,22]. Moreover, secondary hemochromatosis can be also caused by genetic disorders such as beta thalassemia especially if patients have received a large number of blood transfusions [23]. Many types of iron overload, other than the transfusion-linked hemochromatosis, are likely to be associated with diet- or other external factor-linked causes, such as dietary iron overload via consumption of high iron-containing food, hemolysis-linked iron overload via foodborne factors (infection and intoxication), and stress-impaired iron metabolism, all of which contribute to the disruption of iron homeostasis (Figure 2). The present review will address the diet- and stress-linked etiologies of secondary hemochromatosis and their mechanistic evidence in terms of human nutrition and metabolism. In particular, the crosstalk among the genes, nutrients and environment will give novel insights into the understanding of the pathogenesis of secondary hemochromatosis and provide a potential link to chronic complications in patients with hemochromatosis.

## 2. Dietary Iron Overload 

### 2.1. Iron Overload Via Consumption of High Iron-Containing Food

As mentioned, secondary hemochromatosis is due to either iron overload or iron metabolic impairment. In contrast with the blood transfusion-linked hemochromatosis, dietary iron excess tends to increase the systemic levels of both heme and nonheme irons, including circulating ferrous ion in some populations. In particular, it is common in sub-Saharan African populations who have the custom of drinking a fermented beverage with high nonheme iron content [24,25,26]. Dietary iron overload is more common in men than women, while the prevalence and severity increases with age [27]. As with hereditary hemochromatosis, different liver pathogenic processes, including hepatic portal fibrosis, micronodular cirrhosis and hepatocellular carcinoma (HCC), are notable sequelae of the dietary iron overload since the liver is the organ that is most likely to be inflicted by circulating iron [28,29,30]. In terms of histological patterns, the nonheme iron deposition shown in the African population is prominent in both cells of the mononuclear–phagocyte system and hepatic parenchymal cells, whereas hereditary hemochromatosis generally does not display elevated iron accumulation in the macrophages [27]. An exception concerns patients with ferroportin disease caused by mutations of the solute carrier family 40 member 1 gene (*SLC40A1*), who also display iron overload, primarily in Kupffer cells and other macrophages [31]. In addition to the hepatic lesions, dietary hemochromatosis has been linked to the development of metabolic disorders, including type 2 diabetes, chronic kidney disease and cardiomyopathy, using the experimental models [32,33,34]. In addition to the direct toxic actions of iron excess, the metabolic and inflammatory factors can mediate the age-linked pathological aggravation, which may contribute to the sequelae of the hemochromatosis. Although the mechanistic evidence still needs to be addressed, iron-induced oxidative/nitrative stress and reduced antioxidant capacity play important roles in mediating the pathologic events in the complications of dietary hemochromatosis. Moreover, nutritional iron overload-linked pathological patterns and complications in humans are similarly verified in the animal exposure model of dietary iron overload, supporting oxidative stress-associated disease severity [34]. 

### 2.2. Gene–Nutrient–Environment Interactions in Hemochromatosis

All clinical outcomes in African iron overload cannot be explained only by dietary factors. Since not all black Africans that consume large volumes of iron-rich fermented beverage have accumulated iron in the liver [24,25,26], it is expected that a genetic predisposition plays a role in the pathogenesis of African dietary hemochromatosis. The interaction between iron and genes can be implicated in a polymorphic variant (Gln248His) of the FPN-1-endocing *SLC4A0A1* gene in African-Americans with their propensity to develop iron overload [35]. Although this variant was not yet identified in sub-Saharan African populations, it can suggest a potential crosstalk between genetic factors and dietary iron overload. Although typical patients with symptomatic iron overload have normal liver condition without alcoholism or viral hepatitis [15], intrahepatic iron overload by the genetic defects can promote the progression of the infective liver injuries. In particular, chronic hepatitis C tends to be aggravated by the presence of heterozygous HFE mutations, which leads to a high deposition of hepatic iron and advanced stages of fibrosis [36,37]. In addition to the viral infection, patients with hereditary hemochromatosis tend to display altered responses to other environmental factors, such as alcohol and smoking [38,39]. Among the non-genetic modifiers of hereditary hemochromatosis, reduced consumption of alcoholic beverages and body weight increase can explain decreased long-term iron load in hereditary hemochromatosis, while tobacco smoking may aggravate iron loading [39]. Mechanistically, these beneficial environmental modifiers can protect the progression of hereditary hemochromatosis-associated injuries in various organs via improved hepcidin production [39]. Animal studies also demonstrated that increased alcohol consumption down-regulates hepcidin mRNA expression, which was counteracted by blocking of alcohol metabolic enzymes [40,41]. Moreover, dietary antioxidants including vitamin E abolished down-regulation of hepcidin transcription [41], suggesting that alcohol metabolism-mediated oxidative stress aggravates hemochromatosis. Another nutritional supplementation with tannins via regular tea drinking is clinically associated with reduced iron absorption and the low frequency of phlebotomies in patients with hereditary hemochromatosis [42]. Whereas environmental factors may aggravate or ameliorate the progression of hereditary hemochromatosis, the only environmental or non-pharmacological interventions that are successfully proven are dietary restriction of iron availability and venesection. 

## 3. Hemolysis-Associated Hemochromatosis during Foodborne Microbial Infection and Intoxication

Many biological (infection), chemical (toxins, metals) and physical factors (irradiation) from food may affect iron metabolism and iron-associated pathogenesis, such as hemolysis [43,44,45,46,47]. Heavy metals, including cadmium, mercury, lead, arsenic, manganese, chromium, cobalt, nickel, copper, zinc, selenium, silver, antimony and thallium, can be potential inducers of hemolysis when mammals are exposed to these toxic metals via circulation [48,49,50]. In particular, insufficient erythropoietin production and tissue iron accumulation are the central events in cadmium-induced hemolysis [49]. Although the mechanism of heavy metal-induced hemolysis is controversial, the increase of peroxide radicals play crucial roles in disruption of the RBC membrane [51]. Moreover, heavy metals lead to glutathione depletion [52] or disturbance in radical-metabolizing enzymes, such as superoxide dismutase, catalase or glutathione peroxidase [52,53], causing severe oxidative stress-associated massive hemolysis and subsequent hemochromatosis. 

Foodborne infections and bacterial toxins also trigger massive hemolysis. Hemolytic uremic syndrome is a representative toxicoinfection by Shiga toxin-producing bacteria, such as enterohemorrhagic *Escherichia coli* and several *Shigella* species [54,55]. Shiga toxins can induce microangiopathic hemolysis by the activation of vascular endothelial cells, platelet and complement system [56,57]. Among them, the pathogenic events in the vascular tissues play key roles in determining the disease severity. There are two groups of Shiga toxins, which are namely, Shiga toxin type 1 and type 2 (Stx1 and 2) [58], which translocate to the ribosome via the retrograde translocation and specifically bind to and stall the ribosome during translation. The translational inhibition of global proteins in the ribosome activates the integrated stress responses, leading to various pathologic events, such as inflammation and sepsis [59,60,61]. In addition, some other microbial ribotoxins that include trichothecenes from grain-based foodstuff contaminated with the toxigenic molds, such as *Fusariun* species, are known to induce hemolysis [46,62,63]. Depending on the histological and toxicokinetic susceptibility, the fungal trichothecene mycotoxins, including T-2 toxin and deoxynivalenol, can induce differential levels of hemolysis in mammalians [46,63,64]. As implicated in the bacterial ribotoxins, an acute high level of exposure to the fungal trichothecenes produces the radiomimetic syndrome in patients acutely exposed to toxin-contaminated food [65,66]. In response to these foodborne infection or intoxication, the pathologic events such as leukopenia and hemolysis may discharge heme into the blood circulation, resulting in the accumulation of an excessive amount of iron ions in many organs. Foodborne infection- or intoxication-linked hemolysis as a cause of hemochromatosis can increase the systemic levels of irons in the circulation, which increases the risk of iron deposition and toxicities in a broad range of tissues. In addition to hemochromatosis via hemolysis, ribotoxins can cause hemolysis-independent disruption of iron metabolism, which will be discussed in Section 4.

## 4. Stress-Impaired Iron Metabolism

### 4.1. Hepcidin-FPN Axis as the Environmental Sentinel

As mentioned earlier parts, hepcidin is a key regulator of dietary iron uptake in response to systemic iron status, In particular, the hepatic hepcidin is the main negative modulator of the posttranslational control of FPN-1 in the liver–target organ axis [67,68,69]. Hepcidin is a peptide hormone that is encoded by HAMP gene and is a key regulator in iron homeostasis [70]. The hepcidin level is increased by increased iron loading, which is a homeostatic response of the body to restrict iron absorption. However, abnormally high levels of hepcidin under the pathologic conditions lead to anemia due to iron deficiency. In contrast, dietary iron deficiency decreases hepcidin production, leading to intestinal iron absorption and iron release from macrophages so that more iron is available for the body’s needs [10,71,72]. In response to external insults, such as infections, the hepcidin–FPN axis provides an antimicrobial machinery by blocking the iron supply to the blood vessel, which is available for microbial growth [73]. Likewise, other environmental factors, including metals, can disrupt the hepcidin–FPN axis and induce hemochromatosis [74,75,76]. Moreover, FPN1 expression can be transcriptionally regulated by iron and other transition metals. For instance, metals, such as zinc and cadmium, can activate the metal response element-binding transcription factor-1 (MTF-1), which can promote the expression of FPN-1 and metal efflux from the cells for protection [76]. As a counteracting response to metal influx in the circulation, treatment with heavy metals, such as lead, can enhance the production of hepcidin and consequent sequestering of splenic irons, resulting in reduced iron availability for erythropoiesis and metal-induced hemolytic anemia in severe cases [50]. Mechanistically, FPN-1 is internalized without phosphorylation in response to hepcidin binding [77]. Hepcidin-induced endocytosis of FPN-1 is highly dependent on ubiquitination in lysine residues of FPN-1 [78] Internalized FPN-1 cannot be available for iron export from intracellular space to the circulation. Various environmental factors, such as infections, may influence hepcidin expression and subsequent hemochromatosis in the extrahepatic regions, including the brain. For example, bacterial lipopolysaccharide induces the hepatic hepcidin production, which downregulates the bioavailable FPN-1, leading to intracellular iron accumulation in the brain [79,80]. Perturbations in iron homeostasis and iron accumulation are observed in the neurodegenerative disorders, including Alzheimer’s disease and Parkinson’s disease [79]. When taken together, the hepcidin–FPN axis is a crucial sentinel in response to internal and external stressors, including infection, inflammatory or inorganic toxic insults. Disruption in this axis of sentinel leads to hemochromatosis. 

### 4.2. IRP/IRE System as the Environmental Sentinel

The iron responsive element binding protein (IRP) binds to the iron responsive element (IRE), a short conserved stem-loop cis-element. This protein is crucial in iron metabolism because of the translational regulation of ferritin and transferrin receptor (TfR), which is needed for the import of iron into the cell [81,82]. IRP is the functional complex that consists of IRP1 and IRP2 as the Fe-S cluster assembly, which binds to the 5′- and 3′-untranslated regions (UTRs) of the mRNA of target genes. Iron deficiency allows the regulatory action of IRP. Binding of IRP to the 3′-UTR stabilizes TfR1 mRNA whereas binding to the 5′-UTR halts translation of the mRNAs of FPN-1 and ferritin. In contrast, excess iron ions bind to F-box/LRR-receptor protein 5 (FBLX5) and induce protein degradation of IRP via proteasomal activation, leading to induction of ferritin and reduction of TfR [83,84]. Depending on the relative location of IRE and the open reading frame, the translation will be differentially regulated [85]. When cellular iron is scarce, IRP molecules are available for binding to the 5′-IRE and the initiation of translation of ferritin or ferroportin 1 is blocked. In contrast, when 3′-IRE is occupied by available IRPs, this enhances the stability of the TfR transcript. When iron is abundant, very few IREs are occupied by IRPs and TfR mRNA is rapidly degraded, but more ferritin translation occurs [81]. Ultimately, iron excess downregulates TfR-mediated iron acquisition, while promoting ferritin-mediated storage and FPN-mediated export. Therefore, ferritin is a representative marker of iron overload diseases, such as hemochromatosis. Furthermore, the IRP/IRE system also regulates the level of ferritin mRNA, which is responsible for iron storage in cells and works as a buffer for iron deficiency. Because ferritin stores iron ions in a non-toxic form, the degradation of ferritin mRNA can aggravate hemochromatosis-related pathologies. Although ferritin can be used as a potential marker of hemochromatosis, some of inflammatory or metabolic stressors can elevate ferritin levels irrespective of iron overload. For example, ferritin levels are high in patients with infections, such as pulmonary tuberculosis [86,87]. Moreover, ferritin can be a potential biomarker of metabolic stress. Ferritin levels throughout childhood are positively associated with cardiometabolic risk in adolescence [88]. In addition to the metabolic diseases, some degenerative disorders display abnormal iron metabolism of IRP-ferritin, which can be associated with oxidative stress during prion infection [89,90]. However, activation of IRP by *Salmonella* infection can promote host innate immunity by inducing expression of antimicrobial proteins such as lipocalin 2 [91]. Moreover, dietary supplements such as phytochemicals can modulate hemochromatosis. Exposure to dietary curcumin known as a biologically active iron chelator perturbs all parameters of iron metabolism, particularly in mice that are fed a low-iron diet and when the animals display a phenotype of iron deficiency anemia, including a decline in serum iron and decreased transferrin saturation [92]. Iron chelation is important for managing patients with iron overload since returning tissue iron levels to normal levels attenuates iron excess-related toxicity. In particular, curcumin treatment activates IRP and TfR1 while downregulating ferritin and hepcidin levels in liver or hepatocytes without a consequence of gastrointestinal toxicity [92,93]. Therefore, although the phytochemical curcumin can facilitate the development of anemia in patients with marginal iron status, it can potently contribute to intervention with hemochromatosis. Taken together, the environmental cue-induced alteration of IRP/IRE system is a hallmark of some inflammatory and metabolic diseases as well as hemochromatosis. However, the disruption of IRE/IRP-linked iron metabolism may aggravate the disease progression. 

### 4.3. NRF/ARE System as the Environmental Sentinel

Although the posttranslational regulation of FPN-1 has been extensively studied, the transcriptional regulation by nuclear respiratory factor (NRF) also plays a crucial role in iron regulation [94]. As transcription factors, NFR1 and NFR2 bind to ARE and regulate the expression of target genes, including cytoprotective factors, such as NAD(P)H quinone dehydrogenase 1, heme oxygenase 1 or Glutamate—cysteine ligase catalytic subunit and FPN-1 [94,95,96]. Usually, NRF2 that is bound to Kelch ECH associating protein1 (KEAP1) cannot bind to the antioxidant responsive element (ARE) and the hijacked NRF2 is degraded through the proteasomal pathway. Oxidative or electrophilic stress stimulates KEAP1, which releases NRF2 that becomes available for a heterodimer formation with small musculoaponeurotic fibrosarcoma (sMAF). Following this, the NRF2/sMAF heterodimer is transported into nucleus and binds to ARE for gene induction [97,98]. Various environmental factors produce oxidative stress, which would modulate NRF2/ARE-linked biological events. For example, ochratoxin A, a type of toxin produced by *Aspergillus ochraceus*, can block the NRF2 pathway in various ways. Foodborne oncogenic ochratoxin A (OTA) can block the translation of NRF2 through miR-132 in LLC-PK1 cells [99,100,101]. As a result, chronic exposure to OTA depletes the protein pools of NRF2 in cells. Besides, OTA also interferes with the translocation of NRF2 into nucleus, the nuclear accumulation of NRF2 under the oxidative stress and the binding of NRF2 to ARE [100,102,103]. Moreover, ribotoxic stress downregulates NRF2 through p38 MAPK, leading to the suppression of FPN 1 in human enterocytes *in vitro* and nematode gut [47]. Murine genetic ablation models demonstrate that regulation of NRF-linked sentinel have potential to contribute to iron metabolism and hemochromatosis. [104,105]. 

In addition to involvement in FPN1 induction and iron efflux, NRF2-ARE pathways can be crucial in ameliorating tissue injuries such as oxidative stress during hemochromatosis. Extended exposure to ribotoxic stress enhances NRF2-linked protection against the oxidative stress in the murine liver and placenta during the gestational period in mice [106,107]. Moreover, the perylene quinone-type mycotoxins trigger a concentration-dependent increase in NRF2-ARE-dependent promoter activity for genes of antioxidant enzymes [108]. In addition, several dietary phytochemicals that are widely distributed in fruits and vegetables can induce NRF-mediated antioxidant and detoxification enzymes in a variety of mode of actions, including Keap1-dependent and Keap1-independent cascades and epigenetic pathways [109]. Therefore, diet-linked hemochromatosis and related disorders can be potentially counteracted by antioxidant responses of natural NRF modulators. Therefore, the ultimate risk of diet-linked hemochromatosis needs to be carefully assessed based on food composition and the complex interaction between components with opposite regulatory action in the NRF/ARE-linked sentinels.

### 4.4. Gut Microbiota, a Crucial Mucosal Sentinel of Hemochromatosis

Iron homeostasis is the important regulatory factor of bacterial infections, colonization and community in the mucosal ecology. Therefore, patients with hemochromatosis would have altered responses to infection and immunity. In particular, luminal iron levels affect the composition of gut microbiota. The epidemic investigation in the gut iron-overloaded population shows a significant reduction in the beneficial *lactobacilli* and increased levels of enteropathogens, such as *Escherichia coli* and *Salmonella* species [110]. As described in the environmental sentinel of the hemochromatosis, the IRP2 and Hfe are important regulators of iron homeostasis and their mutations are closely associated with hereditary hemochromatosis. In the murine model, a deficiency in IRP2 elevates fecal iron concentrations, which may determine the abundance of some gut bacteria [111]. Although *Lactobacillus (L.) murinus* and *L. intestinalis* are highly abundant in Irp2−/− mice, *Enterococcus faecium* and a species similar to *Olsenella* are highly abundant in Hfe-/- mice. Moreover, *in vitro* evaluation suggested that that the iron supplementation increases the growth rate and health benefits of some lactobacillus strains [112]. However, these experimental results are in contrast to the epidemiological evidence on the suppressive effects of hemochromatosis on the beneficial lactobacillus. Thus, there remains the need to address the complicated mechanisms of iron overload-induced dysbiosis between the genetic and environmental etiologies. In terms of intervention, dietary factors are very important in improving the abnormal composition of microbiota and detrimental metabolism by dysbiosis in patients with hemochromatosis. In addition to controlling a high iron content diet, microbiota-targeted nutritional interventions could be another innovative opportunity to improve hemochromatosis. 

## 5. Chronic Predisposing Factors 

From the analyzed evidence, direct nutritional iron overload and hemolysis-inducing foodborne factors appear to substantially contribute to most cases of clinical outcomes of hemochromatosis. However, the stress-impaired iron metabolism is another considerable risk factor of secondary hemochromatosis. Moreover, the stress-responsive sentinels leading to hemochromatosis are also involved in various types of chronic diseases inflammatory, fibrogenic and oncogenic disorders [113,114,115,116]. Conversely, environmental sentinel-linked aggravation of iron metabolism may predispose patients with chronic diseases to hemochromatosis although they have not experienced excessive exposure to external iron and hemolysis.

**Figure 3:**
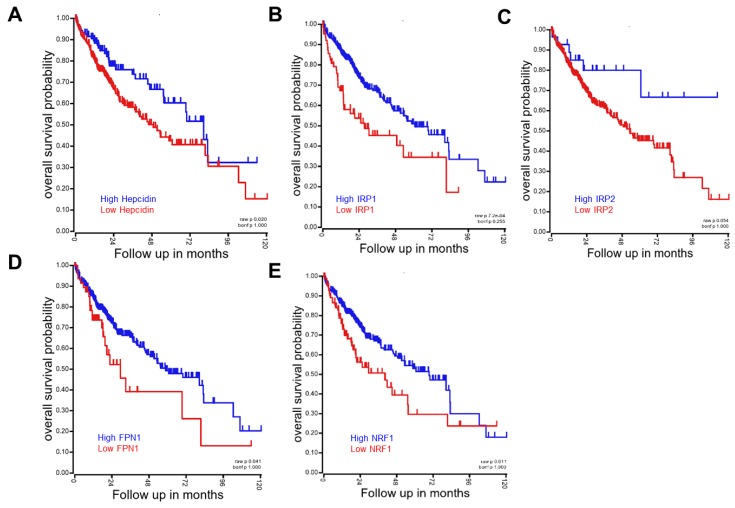
Examples for roles of iron stress sentinels in hepatocellular carcinoma (HCC) survival. The Kaplan–Meier survival plots based on the expression of genes (Hepcidin (cutoff=395.45, (**A**)), IRP1 (cutoff = 2167.24, (**B**)), IRP2 (cutoff = 1563.70, (**C**)), FPN (cutoff = 2757.82, (**D**)) and NRF1 (cutoff = 6179.32, (**E**)), which were obtained from tissues in the patients with HCC (TCGA-LIHC, *n* = 371). This was generated by the Cancer Genome Atlas (TCGA) Research Network: http://cancergenome.nih.gov/. NRF2 did not display significant patterns of gene-associated survival in the present dataset.

Various reports have suggested that patients with hemochromatosis are also susceptible to the development and progression of these chronic diseases, including metabolic diseases, cancer and inflammation [117,118,119]. Untreated hereditary hemochromatosis can be associated with considerable morbidity due to liver cirrhosis, arthritis and diabetes mellitus and increased mortality [120]. The association of hemochromatosis with type 2 diabetes is mechanistically linked to β-cell dysfunction and apoptosis, based on the experimental model using Hfe knockout mice, which is mediated by elevated oxidative stress [121]. Limited studies have linked hemochromatosis to hepatic and extrahepatic malignancies, including esophageal cancer, colorectal cancer, malignant melanoma and lung cancer, despite the conflicting evidence [122,123,124,125,126]. Iron reduction by phlebotomy not only decreased visceral cancer risk by 35% but also decreased mortality in cancer patients by 60% in a supposedly normal population with peripheral arterial disease [127]. As mentioned in Section 2, dietary iron overload in black Africans has been positively associated with progression of the hepatocellular carcinoma (HCC). The risk of HCC development is 4.1 in black patients in southern Africa with dietary iron overload relative to individuals with normal iron status after adjusting for the confounding factors, such as alcohol consumption, hepatic viral infections, cirrhosis and dietary exposure to hepatocarcinogenic aflatoxin B1 [128]. It was also consistent with other observations in Africans that consider the confounding effects of viral infections [129,130]. Among cancers, HCC is one of the most susceptible tumors to iron disorders, which demonstrates the roles of the iron-linked sentineling pathways in the cancer prognosis based on the clinical gene profiling in HCC patients using a public dataset (Figure 3). Regardless of their mode of action in iron metabolism, a lower level of expression of sentineling pathway-linked genes (hepcidin, ferroportin, IRP1/2 and NRF1) was associated with worse prognosis in HCC, which indicates the regulatory actions of the sentineling pathways against the tumorigenesis. Consistent with the analysis in HCC, high ferroportin is a strong and independent predictor of good prognosis of patients with the breast cancer [131]. Thus, all of this evidence suggests that disruption of these iron-linked sentineling pathways is supposed to be detrimental to cancer patients. However, experimental evidence indicates the positive association between iron overload and oncogenic processes, including tumor initiation and promotion. Mechanistically, the local deposition of an excess amount of iron induces toxicity via oxidative radical production and peroxidation of lipids and DNA in cells [132,133]. Furthermore, lipid peroxidation can induce the activation of growth factors, such as TGF-β1, which is a driver of fibrosis through enhanced collagen accumulation and organelle damage [134]. Although the most well-known mediator between iron overload and chronic diseases, including tumors, is oxidative stress, additional mechanistic evidence needs to be identified in the biological network that leads to the redox disturbance. Taken together, while the iron overload can promote tumor initiation and progression, the iron-linked sentinels can exert regulatory actions against the malignancy-associated outcomes in patients. However, systematic investigations and tracking of diverse phases in secondary hemochromatosis are further warranted for better understanding of the interplay between gene and nutrients via stress sentineling pathways in the malignant complications. 

## 6. Conclusions

In addition to the direct dietary iron exposure, various disease states, including infection, hemolysis and chronic distress, disturb iron homeostasis, which is comprehensively associated with sporadic cases of hemochromatosis. Although the mechanistic links could be complicated, the stress-responsive sentinels support the prediction or monitoring of the outcomes of nutritional hemochromatosis in association with genetic mutations. Many metabolic, inflammatory and other pathologic insults can alter the stress sentineling pathways, leading to hemochromatosis, irrespective of external iron overload. While the stress sentinel-linked molecular events mediate the initiation and progression of secondary hemochromatosis, mutations in these sentineling pathway-linked genes are also involved in hereditary hemochromatosis. Therefore, the crosstalk between the hereditary and environmental etiologies via stress sentineling need to be assessed in order to understand the complex procedure, which leads to the clinical outcomes of hemochromatosis. Environmental stress sentinels and other external factors provide potential clues for secondary hemochromatosis and associated complications. Based on mechanistic evidence, more comprehensive and integrated clinical or nutritional interventions need to be developed.
